# Diversity of antibiotic resistance genes in soils with four different fertilization treatments

**DOI:** 10.3389/fmicb.2023.1291599

**Published:** 2023-10-20

**Authors:** Zhuoran Wang, Na Zhang, Chunming Li, Liang Shao

**Affiliations:** ^1^Key Laboratory of Groundwater Resources and Environment of Ministry of Education, College of New Energy and Environment, Jilin University, Changchun, China; ^2^Jilin Provincial Key Laboratory of Water Resources and Environment, College of New Energy and Environment, Jilin University, Changchun, China; ^3^National and Local Joint Engineering Laboratory for Petrochemical Contaminated Site Control and Remediation Technology, Jilin University, Changchun, China; ^4^Jilin Bishuiyuan Water Science and Technology Ltd., Co., Changchun, Jilin, China

**Keywords:** soil properties, antibiotic resistance genes, bacterial community, fertilization, horizontal gene transfer

## Abstract

Although the enrichment of resistance genes in soil has been explored in recent years, there are still some key questions to be addressed regarding the variation of ARG composition in soil with different fertilization treatments, such as the core ARGs in soil after different fertilization treatments, the correlation between ARGs and bacterial taxa, etc. For soils after different fertilization treatments, the distribution and combination of ARG in three typical fertilization methods (organic fertilizer alone, chemical fertilizer alone, and conventional fertilizer) and non-fertilized soils were investigated in this study using high-throughput fluorescence quantitative PCR (HT-qPCR) technique. The application of organic fertilizers significantly increased the abundance and quantity of ARGs and their subtypes in the soil compared to the non-fertilized soil, where sul1 was the ARGs specific to organic fertilizers alone and in higher abundance. The conventional fertilizer application also showed significant enrichment of ARGs, which indicated that manure addition often had a more decisive effect on ARGs in soil than chemical fertilizers, and three bacteria, *Pseudonocardia*, *Irregularibacter*, and *Castllaniella*, were the key bacteria affecting ARG changes in soil after fertilization. In addition, nutrient factors and heavy metals also affect the distribution of ARGs in soil and are positively correlated. This paper reveals the possible reasons for the increase in the number of total soil ARGs and their relative abundance under different fertilization treatments, which has positive implications for controlling the transmission of ARGs through the soil-human pathway.

## 1. Introduction

Antibiotics were first discovered in the 1920s and are widely used to fight bacterial diseases because of their killing or immunosuppressive effects on pathogens (Sarmah et al., 2006; Olesen et al., 2018). Antibiotics are used not only to treat various infectious diseases, but also to ensure medical safety. However, the long-term use of antibiotics can make a large number of disease-causing bacteria resistant to antibiotics, which poses a serious threat to human public health safety, and because the large-scale unregulated use of antibiotics generates important selection pressure for drug-resistant bacteria, making the bacteria in the environment resistant or even multi-drug resistant, causing a series of environmental problems (Zhou et al., 2013; Zhi et al., 2019). It was reported that livestock accounted for 52% of the 196,000 tons of antibiotics used in China, with non-medical use accounting for 70% of all veterinary use (Liu et al., 2022). Due to the slow absorption and low degradation rate of antibiotics in animals, only 10% can be absorbed and utilized, and most of the antibiotics used in excess will be excreted in their original form or metabolites through urine or feces, and then enter the water, soil and air, causing environmental pollution (Karci and Balcioglu, 2009; Zhang et al., 2015). The sources of ARGs include both intrinsic resistance and exogenous inputs (Boxall et al., 2006). Among them, an important source of ARGs production in soil is exogenous input. The main use of antibiotics as drugs to treat infections caused by pathogens is to protect human health and promote the healthy development of animal husbandry (Lillenberg et al., 2010), but excessive use is not only not absorbed and utilized by humans and farm animals, but also excreted out of the body with manure and eventually into the environment. Antibiotics in the environment can put stronger selection pressure on indigenous microorganisms, leading to the development of antibiotic resistance (Karkman et al., 2019; Xu et al., 2022).

Antibiotic resistance genes (ARGs) are genetic elements carried by microorganisms that cause them to develop resistance to target antibiotics (Fu et al., 2022). They are persistent and are the source of bacterial drug resistance. After the death of microbial strains carrying ARGs, the DNA carrying ARGs is released into the environment and persists for a long time under the protection of deoxyribonuclease (DNAse). Eventually, this exposed DNA in the environment can be transferred into other microbial strains at the genetic level (Hill and Top, 1998). ARGs are difficult to disappear once they are produced, and even if antibiotic selective pressure disappears, ARGs will still persist in microbial populations (Crecchio et al., 2005). While soil is considered as a huge reservoir of ARGs in the natural environment, human activities such as irrigation with reclaimed water and manure application have greatly increased the antibiotic resistance of soil (Salyers and Amábile-Cuevas, 1997; Mustafa and Scholz, 2011), but soil antibiotic residues accumulate day by day due to irrigation with antibiotic-containing wastewater and continuous input of livestock manure as organic fertilizer to farmland (Yang et al., 2017). During vegetable cultivation, large amounts of animal manure are also applied to improve soil fertility and yield. Numerous studies have shown that manure application not only inputs exogenous ARGs to the soil, but also promotes the proliferation of indigenous drug-resistant bacteria, thus causing enrichment of ARGs in the soil (Pruden et al., 2006; Gao et al., 2018; Apreja et al., 2022). The application of livestock manure and organic fertilizers is not only the most important way for antibiotics to enter the soil, but also an important source of ARGs contamination in the soil environment (Xiong et al., 2018). Application of organic manure to agricultural soils did lead to a significant increase in the level of microbial antibiotic resistance in the soil (Heuer et al., 2011a). Marti conducted various microscopic and field experiments on agricultural soils to which pig manure, chicken manure, and cow manure were applied and found that the application of all three manures resulted in a significant increase in the abundance of ARGs in the soil (Marti et al., 2013). Schmitt found that tetracycline ARGs (*tetT*, *tetW*, *tetZ*) were commonly present in swine manure and soil, while *tetY*, *tetS*, *tetC*, *tetQ*, *tetH* were also introduced into the soil by manure application (Schmitt et al., 2006). Tang studied rice fields with long-term application of organic fertilizers in the past decades and found that long-term application of organic fertilizers significantly increased the abundance of ARGs in farm soils (Tang et al., 2015). Heuer found that application of manure containing sulfa drugs significantly increased the abundance of sul2 (Heuer et al., 2011b). Ross and Topp’s study also showed that application of cow manure increased the resistance of ARGs in bacteria (Ross and Topp, 2015). Wu detected high levels of tetracycline-based ARGs in soil near pig farms (Wu et al., 2010). All of the above findings suggest that the application of livestock manure and organic fertilizers is not only the most important way for antibiotics to enter the soil, but also an important source of ARGs contamination in the soil environment.

In this study, we set up an inorganic fertilizer group, an organic fertilizer group and an additional conventional fertilizer group (organic fertilizer mixed with inorganic fertilizer) to compare with the control group. High-throughput fluorescence quantitative PCR (HT-qPCR) was used to analyze ARGs in different treatment groups to assess the similarities and differences of total and core ARGs in different treatment groups, and also to determine the bacterial communities and physicochemical properties affecting the composition of ARGs in soil. This result will help to reduce the enrichment of ARGs during fertilization and has positive implications for explaining the mechanism.

## 2. Materials and methods

### 2.1. Experimental design

In this study, samples were collected from a vegetable base in Daxing District, Beijing (N39。66′, E116。57′), and a greenhouse with an area of 488 m^2^ (61 m × 8 m) was selected for a long-term locational trial of eggplant-sweet pepper rotation. The greenhouse was started in spring 2009 with four different fertilizer treatments as: no fertilizer (CK), chemical fertilizer alone (IF), organic fertilizer alone (OF), and conventional fertilizer (MF) (organic fertilizer mixed with chemical fertilizer).

Organic fertilizer is produced by a large commercial organic fertilizer factory in Beijing, the main raw materials are chicken manure and edible bacteria bran, which are powdered substances made by high temperature aerobic fermentation. Three plots were set up for each treatment, for a total of 12 plots. A 50 cm deep partition was set up between each treatment, separated by plastic film to avoid interference between different treatments (Rambaut et al., 2022). The greenhouse grows two crops of vegetables per year, with the eggplant season running from February to July and the bell pepper season running from August to January. One fertilization treatment was applied before each vegetable crop, setting the control group as no fertilizer (CK), the fertilizer alone group (IF) with 0.04 kg/m^2^ of urea, 0.05 kg/m^2^ of diammonium phosphate and 0.03 kg/m^2^ of potassium sulfate, the organic fertilizer alone group (OF) with 2.38 kg/m^2^ (commercial organic fertilizer), and the conventional fertilizer group (MF) with a compound fertilizer (N, P and K content of 20, 10 and 15%, respectively) 0.06 kg/m^2^ and commercial organic fertilizer 1.20 kg/m^2^ in the conventional fertilizer group (MF). The application rates of N, P, K fertilizer were converted from plant uptake amount considering the fertilizer utilization ratio (Cui et al., 2022; Edouard et al., 2022; Li et al., 2022).

### 2.2. Sample collection

The collection of soil samples for this experiment was conducted in October 2019. A 5-point mixed sampling method was used to take the top soil (0–20 cm) of each plot, four different treatments, and three samples were collected from each treatment, for a total of 12 soil samples.

### 2.3. DNA extraction and high-throughput sequencing of bacterial 16S rRNA genes

#### 2.3.1. Soil sample DNA extraction

DNA extraction of soil samples was performed using the kit Fast DNA SPIN Kit for Soil (USA). The instructions were followed. After DNA extraction, the concentration of DNA extracted is measured and a portion of it is used for 16SrRNA sequencing and the rest for PCR quantification and other applications, and stored at −20°C for a long time until use.

#### 2.3.2. High-throughput fluorescent quantitative PCR technology (HT-qPCR)

ARGs were quantified using the Wafergen Smart Chip Real-Time Quantitative PCR System (Wafergen Inc. USA). 384 primer pairs were set (Looft et al., 2012; Chen et al., 2015; Gou et al., 2018), including 348 ARGs, 32 transposase genes, one 16S rRNA gene, and three integrase genes (int1, intl2, and intl3). These 348 primers for ARGs cover almost all known subtypes of ARGs. The primer premix is sprayed on the same chip as the sample premix for subsequent amplification analysis. The thermal cycling process is: 10 min at 95°C, followed by 40 cycles at 95°C for 30 s and 60°C for 30 s. The relative copy number of ARGs can be calculated by the formula (Su et al., 2015) (1).


(1) Relative copy number of genes=10[(26−Ct)/(10/3)]

(Note: Ct value is the number of cycles required for the fluorescence signal of the test gene to reach a set threshold).

### 2.4. Statistical analysis

EXCEL 2013 was used to organize the data, Origin Pro 8.5 was used to draw bar graphs, the pheatmap package in R language was used to draw heat maps, the vegan package was used for redundancy analysis (RDA), and the TukeyHSD method was used for statistical analysis and PcoA principal coordinate analysis. Mantel test and typical correlation analysis (RDA) were used to explain the relationships between heavy metals (As, Hg, Cu, Cr, Pb, Cd, Zn), nutrient factors (organic matter, effective phosphorus, fast-acting potassium, total nitrogen), pH and ARGs. *p* < 0.05 indicates significant differences at 95% confidence interval. Network plots were drawn using Gephi software.

## 3. Results and discussion

### 3.1. Diversity of ARGs in soils

#### 3.1.1. Component analysis of ARGs in soils

**Figure 1:**
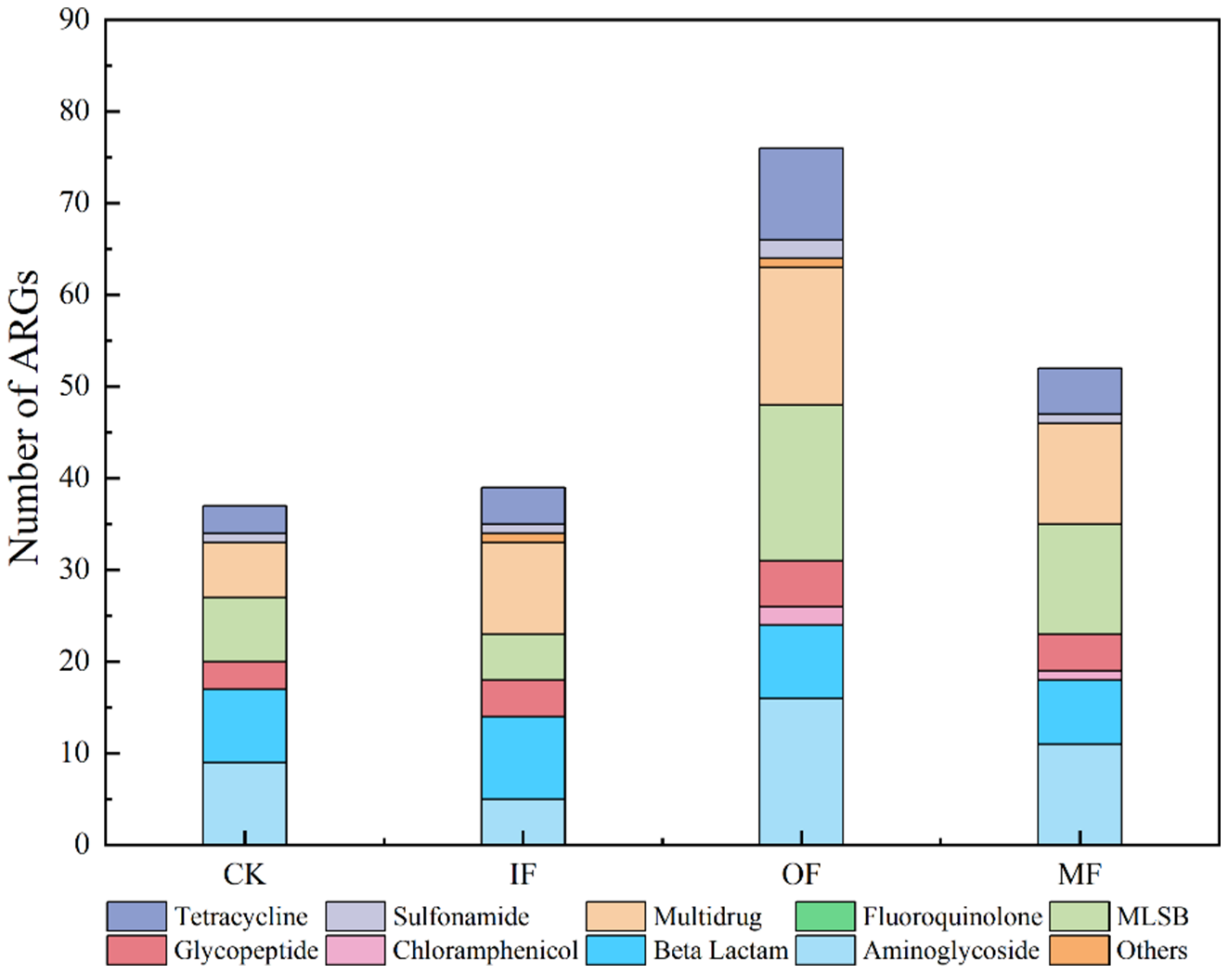
The number of ARGs in the soil amended with different fertilizers.

**Figure 2:**
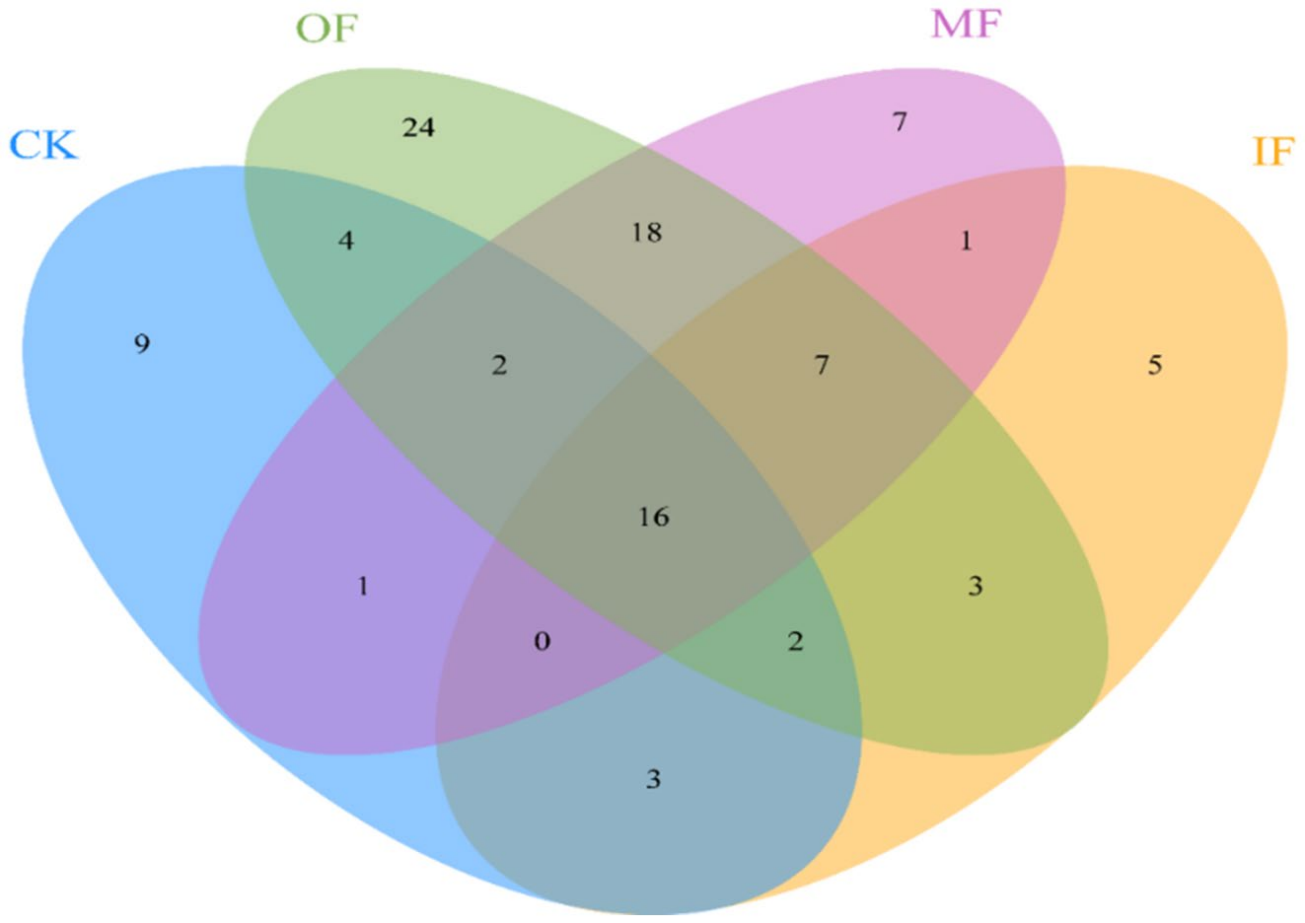
Venn plots of soils treated with different fertilizers.

The number of ARGs subtypes in soils applied with different types of fertilizers was examined, and a total of 37–76 ARGs subtypes were detected in the soils of four different fertilization treatments (Figure 1). They belong to Aminoglycoside, *β*-Lactamase, Chloramphenicol, MLSB, Multidrug, Clycopeptide, Sulfonamide, Tetracycline, Fluoroquinolone, and Others. The highest number of ARGs was 76 in organic fertilized soils, followed by 52 in conventionally fertilized soils, 37 in chemical fertilized soils alone, and the lowest number of ARGs was 36 in non-fertilized soils (Figure 2). There were 16 core ARGs (ARGs common to soils with different fertilization treatments) for no fertilizer, chemical fertilizer alone, organic fertilizer alone and conventionally fertilized soils, with β-lactams accounting for the largest number, 37.5% of the total purpose, and multiresistant and sulfonamides the least, both at 6.25%. There were 9 subtypes of ARGs specific to the no-fertilizer treatment, the largest number being aminoglycosides (*aac*, *aac(6′)-Ib(aacA4)-01*, *aadA5-01*, *aph*), 5 fertilizers alone, with the same number accounted for by different types, and 24 organic fertilizers alone, with the largest number being macrolides (*erm(36)*, *ermB*, *ermF lnuA-01*, *lnuB-01*, *lnuB-02*), followed by tetracyclines (*tet(32)*, *tetM-01*, *tetM-02*, *tetPB-03*, *tetX*) with aminoglycosides (aminoglycosides *aacA*, *aadA-1-01*, *aadD*, *aph(2′)-Id-02*, *str*), and 7 conventional fertilizers. The most multidrug-resistant classes (*ceoA*, *marR-01*, *mexE*). The total number of ARGs in the soil with organic fertilizer alone was approximately twice as high as that in the soil without fertilizer and the specific ARGs subtypes were much higher than those in the other three treatment groups, indicating that organic fertilizer alone increased the diversity of soil ARGs. In addition, for soils with mono-applied organic fertilizers, Aminoglycoside and MLSB ARGs were the most abundant, followed by Multidrug, which were largely consistent with the most numerous subtypes of endemic ARGs in mono-applied organic fertilizers. And it has been reported in the literature that long-term application of chicken manure significantly enriched the ARG associated with *β*-lactams and tetracyclines in the soil, which is consistent with the results obtained from the application of organic fertilizer alone in this study, where there was a significant increase in the number of *β*-lactam and tetracycline-based resistance genes in the OF group (Chen et al., 2016). Interestingly, the total count of ARGs in non-fertilized soil varied by merely one ARG subtype in comparison to that of the fertilized soil alone, which also augmented the diversity of ARGs, albeit to a vastly lesser extent compared to the soil treated with organic fertilizer alone. This minimal increase could be ascribed to the alterations in the population of natural antibiotic-resistant bacteria in the soil post fertilizer application. There was a significant increase in the variety of ARGs in the conventional fertilizer application compared to the no fertilizer and single fertilizer application groups, although organic fertilizers were also added but still compared to single fertilizer application.

#### 3.1.2. Abundance analysis of ARGs in soils

**Figure 3:**
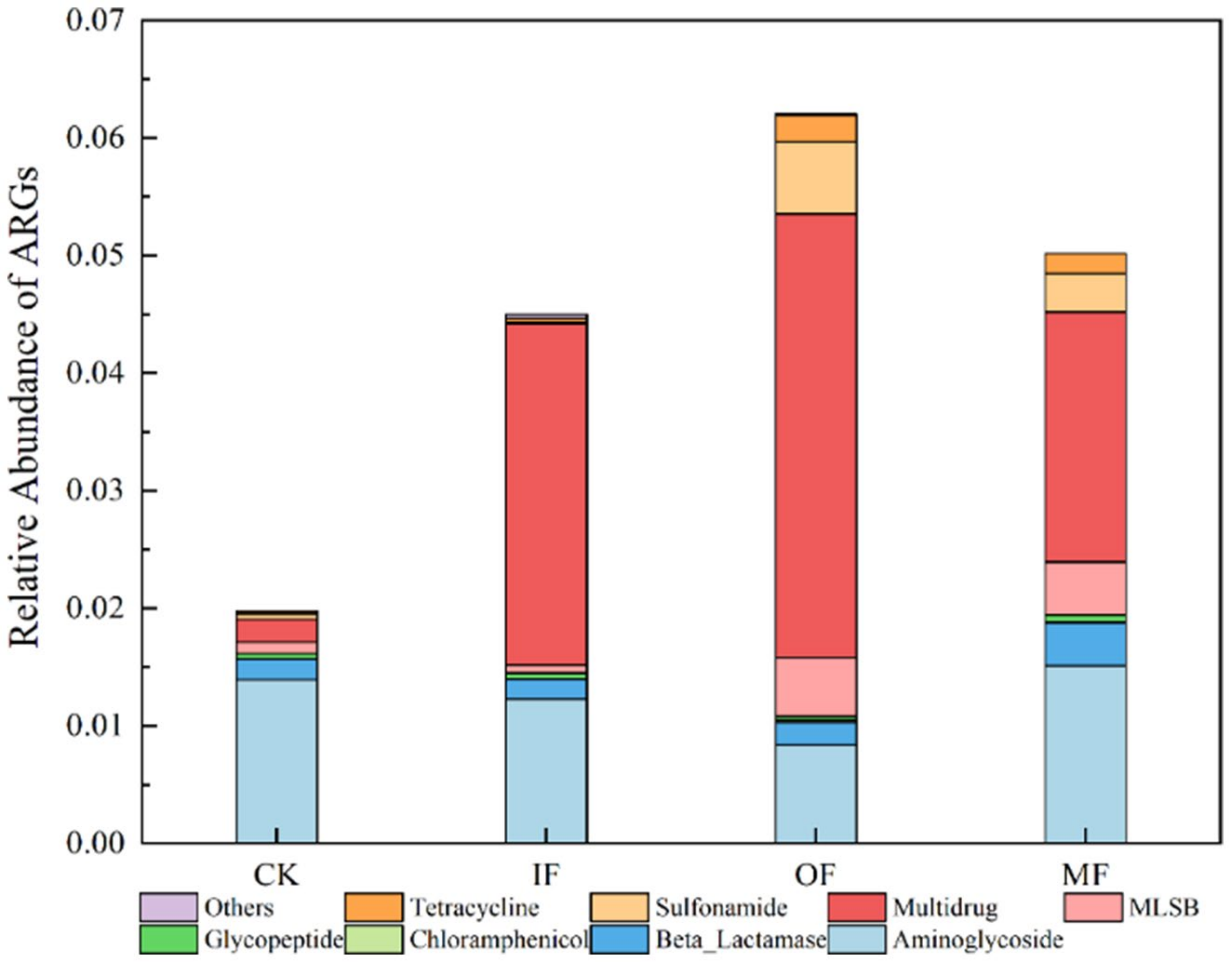
Relative abundance of ARGs in the soil amended with different fertilizers.

Figure 3 represents the relative abundance of ARGs in soils with different fertilization treatments. The results from the figure show that the relative abundance of total ARGs in soils with no fertilizer, chemical fertilizer alone, organic fertilizer alone, and conventional fertilizer were 0.01978, 0.04498, 0.06203, and 0.05016, respectively, with OF > MF > IF> CK. This finding is similar to the previously reported values of ARGs abundance in soils applied with chemical and organic fertilizers. The total relative abundance of ARGs was significantly higher in the organic fertilized soil than in the non-fertilized, chemical fertilized alone and conventional fertilized soils. The relative abundance of ARGs of multiple resistance classes was significantly higher than that of other resistance genotypes in soils without fertilizer, with chemical fertilizer alone and with organic fertilizer alone. It has been reported that microorganisms carrying multi-drug resistant genes may be resistant to a variety of antibiotics and have the potential to develop into “superbugs” which, if not controlled, can increase the lethality of human infections. In addition to multidrug-resistant classes, aminoglycosides, sulfonamides and macrolide ARGs also had high abundance in soils with single organic fertilizer application, and the relative abundance of multidrug-resistant classes accounted for 60% of the total relative abundance, and the highest relative abundance of aminoglycosides ARGs was found in soils without fertilizer application. It was also observed that fertilizer application alone had little effect on the total abundance of soil ARGs and the abundance of different types of ARGs, but the application of organic fertilizer significantly increased the abundance of macrolide and sulfonamide ARGs. Wang also found that the application of organic fertilizers significantly increased the relative abundance and the number of ARGs detected (Wang et al., 2020), and the application of chemical fertilizers had less effect on the relative abundance of ARGs than that of organic fertilizers due to the fact that when ARBs in manure were introduced into the soil, the ARGs they carried could also be transferred horizontally to the soil, whereas when inorganic fertilizers were applied only the native ARG-carrying microorganisms in the soil were proliferated. The utilization of animal feces as organic fertilizer through composting is a common practice. Following the composting process, a notable augmentation in the abundance of *Bacillus* spp. is often observed in the soil. It has been established that *Bacillus* spp. serve as host bacteria for resistance genes associated with tetracyclines, sulfonamides, and β-lactams, among others. In synthesis, the remarkable increase in the abundance of ARGs post organic fertilizer application can be ascribed to the substantial influx of *Bacillus* spp. into the soil via composting. The ARGs have the potential to be transferred horizontally to other media, thereby leading to a significant augmentation in both the abundance and diversity of ARGs within the soil. As a host for a myriad of ARGs, *Bacillus* spp. consequently lead to a significant rise in ARG abundance in the groups treated with organic fertilizer (Qi et al., 2023). The abundance of multi-drug resistant ARGs in soils with organic fertilizer application increased one-fold compared with that in conventionally fertilized soils and 12-fold compared with that in non-fertilized soils, indicating that the application of organic fertilizer seriously affected the abundance of ARGs. In view of the resistance of multi-drug resistant ARGs and their potential hazards, we need to treat them in some way before organic fertilizer application to mitigate their effects on the abundance of ARGs. For example, He added biochar to the application of manure to make the antibiotic resistance genes in agricultural soils gradually dissipate (He et al., 2021).

#### 3.1.3. Distribution characteristics of ARGs in soils

**Figure 4:**
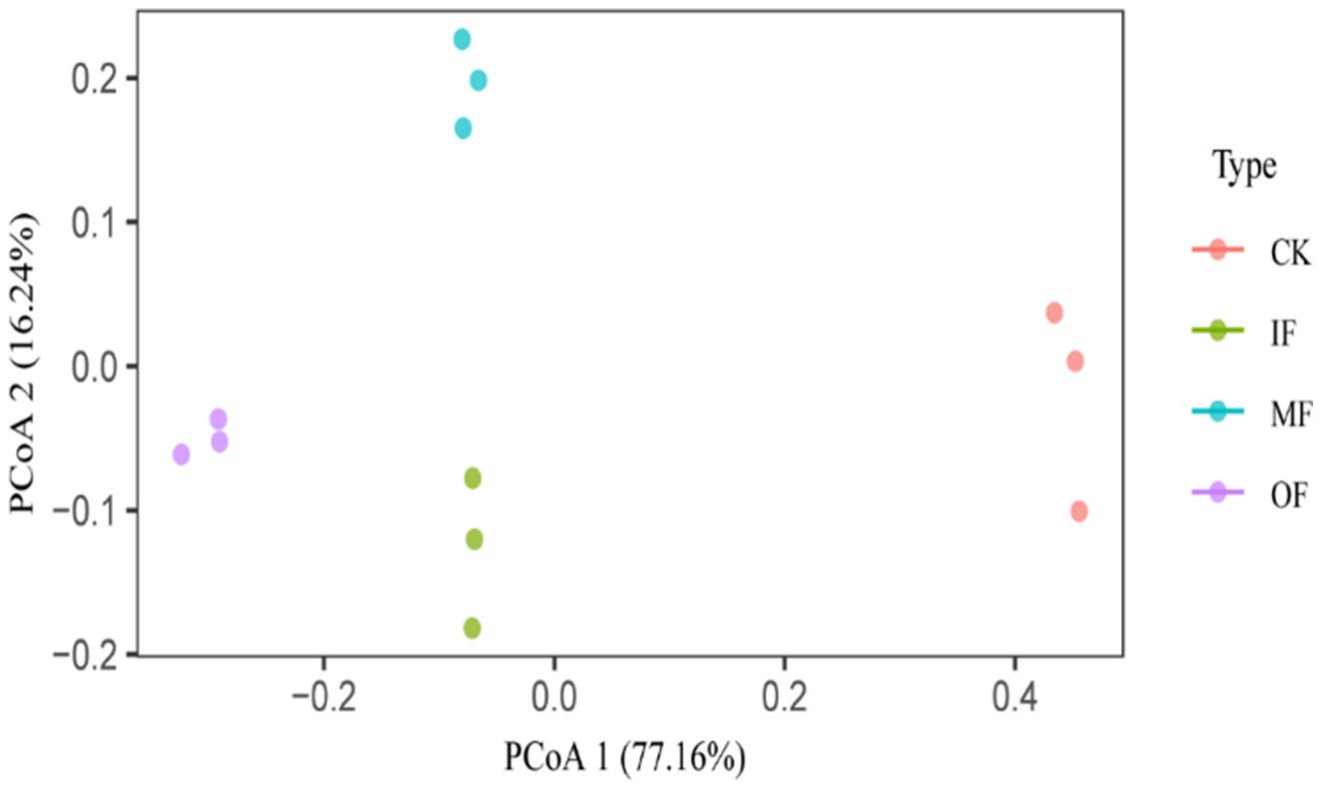
Principal component analysis of ARG in soil under different fertilizers treatments.

To investigate the differences in the characteristics of ARGs in vegetable soils under different fertilization practices, PCoA was performed on the abundance data of ARGs (Figure 4). PCoA1 and PCoA2 explained 77.16 and 16.24% of the variation in the composition of ARGs, respectively. The results of the principal coordinate analysis showed that three fertilization methods, chemical fertilizer alone, organic fertilizer alone and conventional fertilizer, significantly changed the distribution characteristics of soil ARGs (PERMANOVA for treatments: *R*^2^ = 0.9735, *p* = 0.001, Adonis analysis), IF, OF, MF all significantly changed the composition of ARGs in the soil compared to the control. This result further supports the difference in the composition of ARGs in soils with different fertilization treatments.

**Figure 5:**
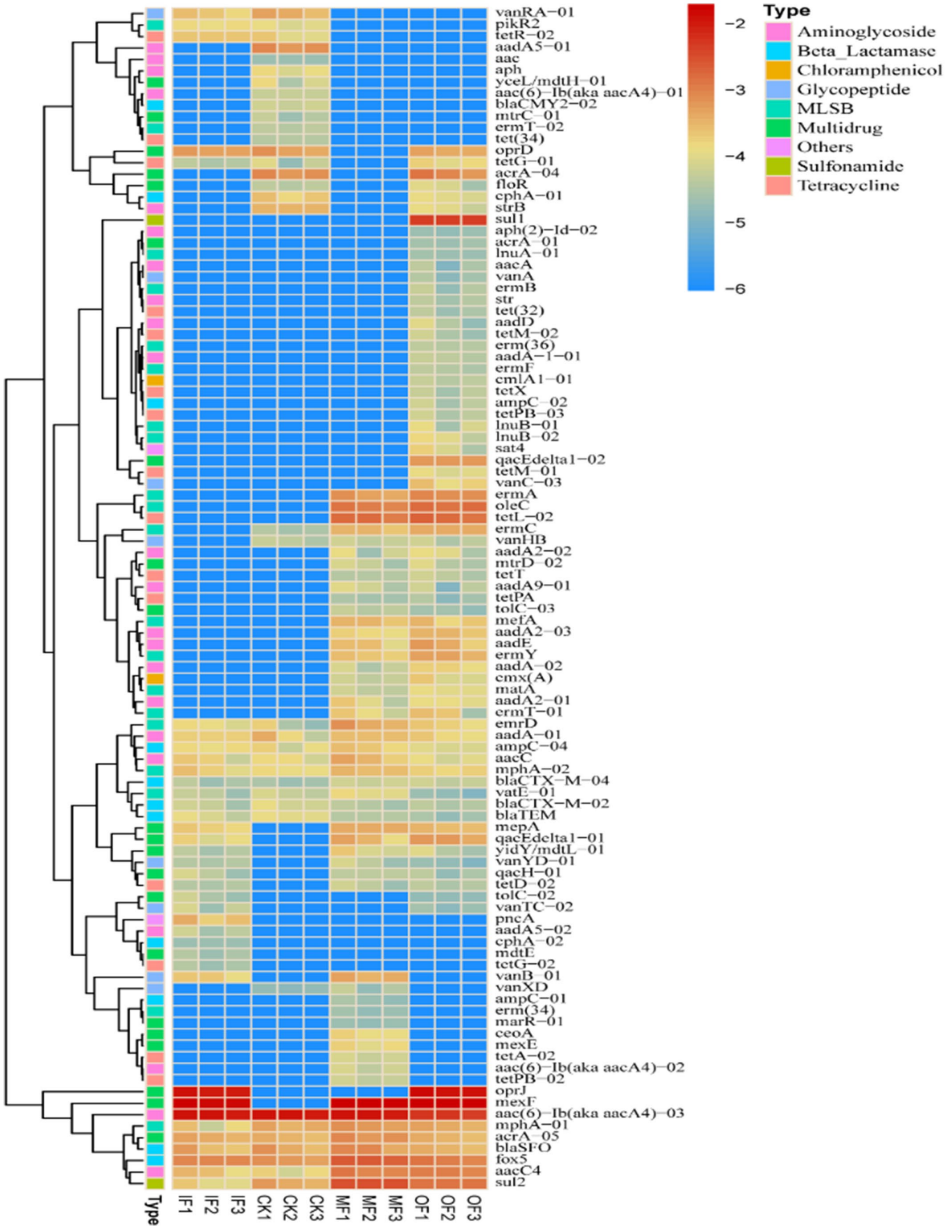
Heat map of ARGs subtypes in soils under different fertilizers treatments.

Figure 5 show heat maps of the relative abundance of ARGs subtypes in soils with different fertilization treatments. The results show that the application of organic fertilizer caused a significant increase in the abundance of most ARGs subtypes in the soil compared to the control soil without fertilizer application. And the application of organic fertilizer significantly increased the abundance of ARGs isoforms such as *mepA*, *qacEdeltal-01*, *acrA-04*, etc. in the soil for multiple resistance classes of resistance genes. There were 24 ARGs specific to the soil with organic fertilizer alone, indicating that the application of organic fertilizer could increase the variety of ARGs in the soil; sul1 (sulfonamides) was the ARGs specific to the soil with organic fertilizer alone, and the relative abundance was high.

The abundance of *vanRA-01* (glycopeptides) was significantly increased in the soil with single fertilizer treatment compared to the soil without fertilizer, and the difference between the shared ARGs in the soil without fertilizer and the soil with single fertilizer was not significant. *oprJ* (multiresistant) was detected only in the soil with single fertilizer and the soil with single organic fertilizer, and its relative abundance was high. *mexF* (multiresistant) subtype had higher abundance in the soil with three different fertilizer applications except for the soil without fertilizer. The high abundance of *mexF* (multidrug-resistant) subtypes in all three fertilized soils, except the non-fertilized soils, indicated that fertilizer application increased the variety of ARGs in the soil. Conventional fertilization had higher relative abundance of *fox5* (*β*-lactams) and *sul2* (sulfonamides) compared to the other three fertilization treatment conditions. The figure also shows that *aac(6)-Ib(aka aacA4)-03* (an aminoglycoside resistance gene) had higher abundance in all treatments, indicating that they have some stability in the soil and should be a key concern.

In addition, *sul2*, a sulfonamide resistance gene, was detected in soils treated with either chemical fertilizer alone, organic fertilizer alone, conventional fertilizer application, and no fertilizer application, and the gene copy number was as high as about 1.44 × 10^−3^ per gram of soil, while sul1 (sulfonamide), a specific ARGs, was detected in soils treated with organic fertilizer alone, and the gene copy number per gram of soil up to 4.72 × 10^−3^, indicating that the application of organic fertilizer changed the type of ARGs in the soil. One study found that *sul2*, a sulfonamide resistance gene, was also detected in 100% of black soil farm soils with organic fertilizer application, which is consistent with the findings of this paper (Yang et al., 2016). Related studies showed that the abundance of sulfonamide ARGs in soil increased significantly after fertilizer application. *β*-lactams, multi-drug resistant, macrolides, and tetracyclines ARGs were detected to varying degrees, and the detection rates were all different, indicating that the application of organic and chemical fertilizers in vegetable soils had a significant effect on these types of ARGs, increasing the subtypes and abundance of ARGs (Schmitt et al., 2006). This result is consistent with the findings of Zhang in vegetable field soils (Zhang et al., 2022). Wu Nan detected the presence of five dominant tetracycline resistance genes *tetB/P*, *tetM*, *tetO*, *tetT*, and *tetW* in soil near a pig farm in Beijing, with the highest level of *tet(W)* 2.16 × 108 copies·g ^−1^ (dry soil), which was 2.89 ± 0.54 × 106 copies·g ^−1^ (dry soil) was about two orders of magnitude higher (Wu et al., 2009). It indicates that either organic fertilizers, chemical fertilizers or mixed application treatments will increase the level of ARGs in the soil. Once the horizontal shift of ARGs in soil occurs, it will definitely have serious impact on the safety of agricultural products and human safety (Riahi et al., 2022).

#### 3.1.4. Correlation analysis of ARGs and physicochemical properties in soils

To assess the correlation between soil ARGs and physicochemical factors, Mantel tests were conducted to determine the abundance of ARGs in vegetable soils with heavy metals, organic matter (OM), total nitrogen (TN), effective phosphorus (AP), and fast-acting potassium (AK) contents as well as pH in four different fertilization treatments. The results showed (Table 1) that heavy metals Cu, Zn, Cd, and As were significantly and positively correlated with ARGs (*p* < 0.01); organic matter, total nitrogen, effective phosphorus, and fast-acting potassium were also significantly and positively correlated with ARGs (*p* < 0.01).

**Table 1:** Mantel test reveals the relationship between ARGs and physicochemical properties.

Factors	Statistic (r)	Sig. (P)
Cu	0.644	0.0037*
Zn	0.867	0.0007*
Pb	−0.044	0.5696
Cd	0.763	0.0011*
Cr	0.084	0.2421
As	−0.146	0.0048*
Hg	0.065	0.2777
pH	0.281	0.0565
TN	0.653	0.0013*
OM	0.770	0.0002*
AP	0.926	0.0002*
AK	0.926	0.0004*

TN, total nitrogen; OM, organic matter; AP, active phosphorus; AK, fast-acting potassium; *significant correlation (*p* < 0.01).

**Figure 6:**
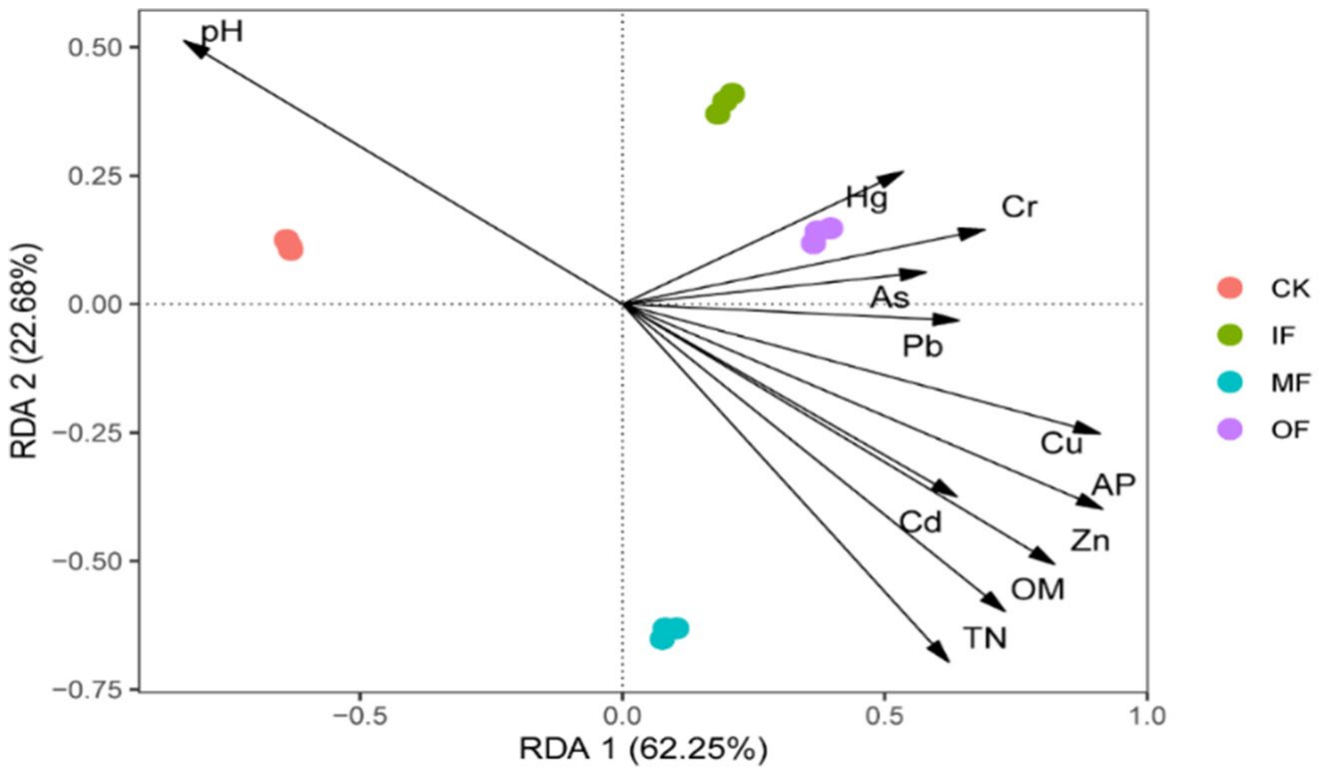
Redundancy analysis of soil ARGs and physical and chemical properties.

RDA was used to further assess the contribution of individual variables to the changes in ARGs, and the results are shown in Figure 6. The results in the figure show that there are 11 environmental factors scattered in four quadrants and mostly clustered in the first and fourth quadrants. The eigenvalues of the 1st and 2nd sorting axes were 62.25 and 22.68%, respectively, which could explain 84.93% of the total variance value of the ARGs variance. Redundancy analysis showed that ARGs in organic fertilized soils were significantly and positively correlated with Pb, Cu, Zn, Cd, total N, organic matter, and effective phosphorus; while pH was significantly and positively correlated with ARGs in control soils, and As, Hg, and Cr were significantly and positively correlated with ARGs in chemical fertilizer alone and conventionally fertilized soils. The utilization of Cu and Zn in soil leads to a rise in the abundance of ARGs, due to the co-selection effect of heavy metals, where the host bacteria of ARGs proliferate alongside heavy metals in the soil, thereby intensifying the horizontal gene transfer of ARGs. Soil physicochemical properties such as pH also play a pivotal role; under alkaline soil conditions, the abundance of ARGs exhibits a positive correlation with pH as it can enhance the activity of other organic matter. Total nitrogen and organic matter, serving as organic substances, supply nutrients to the soil, promoting the proliferation of relevant microbial communities, and thus exacerbating the dissemination of ARGs (Shi et al., 2023).

It can also be seen from the figure that the distribution of ARGs of fertilizer-applied soils clustered with conventionally fertilized soils in the first quadrant, while the ARGs of no fertilizer and organic fertilizer alone were scattered in the second and fourth quadrants, indicating that the application of organic fertilizer significantly changed the distribution characteristics of soil ARGs. In Dong’s study total and effective amounts of Cu and Zn were significantly correlated with *qepA* and *qnrS*. Heavy metals can persist in the environment for a long period of time and increase the abundance of ARGs under sustained selective pressure, and previous studies have also demonstrated that replacing chemical fertilizers with organic fertilizers induces the accumulation of Cd, Pb, Cu, and Zn in the soil, suggesting that fertilizer treatments can influence the abundance of resistance genes associated with them by affecting the accumulation of heavy metals in the soil (Dong et al., 2022; Liu et al., 2023). He studied paddy soils from seven different areas in Sichuan Province and also found a significant positive correlation between total nitrogen and ARGs (He et al., 2020). Other studies have shown that heavy metals and organic matter can promote the persistence and diffusion ability of ARGs in the environment. Therefore, heavy metals and nutrient factors are closely related to ARGs in vegetable soils.

### 3.2. Co-occurrence analysis of ARGs and bacterial communities in soils

**Figure 7:**
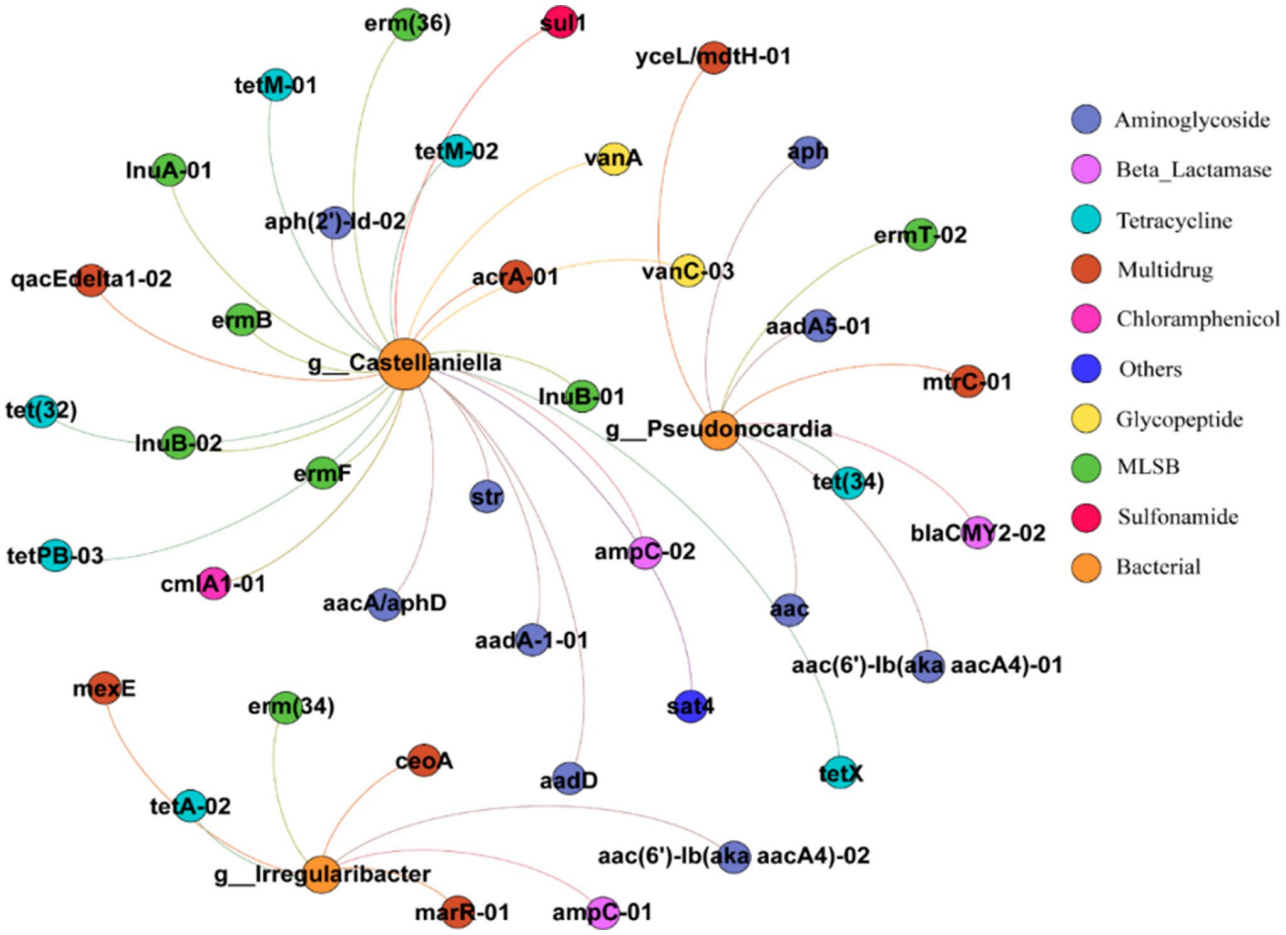
Network diagram of ARGs and bacterial community in soil (circle size represents the total abundance of ARGs and bacteria, node size is weighted according to the number of connections, and one node represents one ARG and bacteria).

The symbiotic relationship between ARGs and bacterial families was investigated using Pearson’s based network analysis and the results are shown in Figure 7 and Qi et al. (2022). Two nodes are connected to indicate the co-occurrence of bacteria and ARGs, and the graph consists of 43 nodes and 40 edges. These nodes contain three bacterial and 40 ARGs subtypes, and the ARGs cover eight classes of resistance gene types. *Pseudonocardia* has 9 lines of association with ARGs (*p* < 0.001), including multiresistant classes, macrolides, *β*-lactams, aminoglycosamides, tetracyclines, and the most subtypes of aminoglycoside ARGs, with 4. *Irregularibacter* has seven linkages with ARGs (*p* < 0.001), including tetracyclines, macrolides, multiresistant classes, aminoglycosides and *β*-lactams, the most numerous of which are the multiresistant classes with three subtypes of ARGs and the other classes with one subtype of ARGs each. *Castllaniella* has 24 linkages to ARGs (*p* < 0.001), which include sulfonamides, chloramphenicol, glycopeptides, and others in addition to macrolides, aminoglycosides, tetracyclines, *β*-lactams, multiresistance, and tetracyclines, and have 1, 1, 2, and 1 subtype of ARGs, respectively. The above results indicate that all three bacteria (*Pseudonocardia*, *Irregularibacter*, and *Castllaniella*) were significantly correlated with ARGs, which is consistent with the previously reported findings that some ARGs were significantly correlated with multiple bacterial taxa (Looft et al., 2012; Su et al., 2015). Correlation analysis revealed that the bacterial genera *Pseudonocardia*, *Irregularibacter*, and *Castellaniella* could potentially serve as host bacteria facilitating the transfer of ARGs. *Pseudonocardia*, commonly found in soil, plants, and various environmental settings, may promote the horizontal gene transfer of a multitude of ARGs (Ejtahed et al., 2020). *Castellaniella*, known to prevail under antibiotic stress, could be a potential reservoir of antibiotic-resistant bacteria, thereby facilitating the horizontal transfer of ARGs within the environment.

## 4. Conclusion

In summary, changes in fertilization practices affect the composition of ARGs in soil, increasing their subtypes as well as abundance. According to our results, although organic fertilizers were also added to the conventional fertilizer application, the increase in the number of ARGs and their abundance was much less than that of the organic fertilizer application alone, while compared to the chemical fertilizer application alone, there was a significant increase in ARGs in the conventionally fertilized soil due to the addition of organic fertilizers. We suggest that the application of organic fertilizers leads to higher number and abundance of ARGs in the soil, and the main reason may be that the ARGs carried in organic fertilizers spread to the soil, while the alteration of ARGs from chemical fertilizer application comes only from the colonization of native microorganisms, and conventional fertilizers are in between. Soil factors TN, OM, AP, AK and heavy metals Cu, Zn, Cd and As contributed more to the variation of ARGs in soil than other factors. These findings provide a more comprehensive insight into the changes of ARGs in soil ecology under long-term application of different types of fertilizers.

## Data availability statement

The original contributions presented in the study are included in the article/supplementary material, further inquiries can be directed to the corresponding author.

## Author contributions

ZW: Conceptualization, Formal analysis, Investigation, Visualization, Writing – review & editing, Writing – original draft. NZ: Resources, Validation, Writing – review & editing. CL: Formal analysis, Writing – review & editing. LS: Investigation, Writing – review & editing.
